# The Antagonistic Effect of Mycotoxins Deoxynivalenol and Zearalenone on Metabolic Profiling in Serum and Liver of Mice

**DOI:** 10.3390/toxins9010028

**Published:** 2017-01-10

**Authors:** Jian Ji, Pei Zhu, Fangchao Cui, Fuwei Pi, Yinzhi Zhang, Yun Li, Jiasheng Wang, Xiulan Sun

**Affiliations:** 1 School of Food Science, State Key Laboratory of Food Science and Technology, National Engineering Research Center for Functional Foods, Jiangnan University, Wuxi 214122, China; jijianjndx@126.com (J.J.); cfc1031@163.com (F.C.); pifuwei@hotmail.com (F.P.); yinzhizhang@jiangnan.edu.cn (Y.Z.); gz-liyun@126.com (Y.L.); 2 State Key Laboratory of Dairy Biotechnology, Shanghai Engineering Research Center of Dairy Biotechnology, Dairy Research Institute, Bright Dairy & Food Co., Ltd., Shanghai 200436, China; peipeiecust@163.com; 3 Synergetic Innovation Center for Food Safety and Nutrition, Jiangnan University, Wuxi 214122, China; 4 Key Laboratory of Agro-Food Safety and Quality of Ministry of Agriculture, Institute of Quality Standards and Testing Technology for Agro-products, Chinese Academy of Agricultural Sciences, No. 12, Zhongguancun South Street, Beijing 100081, China; 5 Department of Environmental Health Science, College of Public Health, University of Georgia, Athens, GA 30602, USA

**Keywords:** deoxynivalenol, zearalenone, combined toxicity, metabolomics, antagonistic effect

## Abstract

Metabolic profiling in liver and serum of mice was studied for the combined toxic effects of deoxynivalenol (DON) and zearalenone (ZEN), through gas chromatography mass spectrum. The spectrum of serum and liver sample of mice, treated with individual 2 mg/kg DON, 20 mg/kg ZEN, and the combined DON + ZEN with final concentration 2 mg/kg DON and 20 mg/kg ZEN for 21 days, were deconvoluted, aligned and identified with MS DIAL. The data matrix was processed with univariate analysis and multivariate analysis for selection of metabolites with variable importance for the projection (VIP) > 1, *t*-test *p* value < 0.05. The metabolic pathway analysis was performed with MetaMapp and drawn by CytoScape. Results show that the combined DON and ZEN treatment has an obvious “antagonistic effect” in serum and liver tissue metabolic profiling of mice. The blood biochemical indexes, like alkaline phosphatase, alanine transaminase, and albumin (ALB)/globulin (GLO), reveal a moderated trend in the combined DON + ZEN treatment group, which is consistent with histopathological examination. The metabolic pathway analysis demonstrated that the combined DON and ZEN treatment could down-regulate the valine, leucine and isoleucine biosynthesis, glycine, serine and threonine metabolism, and *O*-glycosyl compounds related glucose metabolism in liver tissue. The metabolic profiling in serum confirmed the finding that the combined DON and ZEN treatment has an “antagonistic effect” on liver metabolism of mice.

## 1. Introduction

During environmental mycotoxin exposure, humans and animals most often encounter mixtures of toxins, rather than single toxins, such as deoxynivalenol (DON) and zearalenone (ZEN) [1]. Deoxynivalenol (DON)—a representative mycotoxin of the trichothecene B group—is one of the most widespread cereal contaminants worldwide [2]. Numerous studies have addressed the toxicity of DON and its derivatives in animals [3]; swine are the most susceptible species [4,5]. At the cellular level, the trichothecene DON and its derivatives disrupt normal cell function by binding to the ribosome and inhibiting protein synthesis, and by activating cellular kinases involved in signal transduction [6]. Proteome and phosphoproteome strategies were conducted for the interpretation of ribosome functions as a potential platform for spatiotemporal regulation of translation inhibition and ribotoxic stress response caused by DON [7]. The relationship between protein phosphorylation and DONs immunotoxic effects was another research focus [8]. DON could induce glucose-regulated protein 78 (GRP78) degradation and it evokes an endoplasmic reticulum stress response that could contribute, in part, to DON-induced interleukin-6 gene expression [9].

The mycotoxin zearalenone (ZEN) is produced by the *Fusarium* species as well as the metabolites zearalanone, α-zearalanol (α-ZOL) and β-zearalanol (β-ZOL). Both α- and β-ZOL exert harmful heath effect via their strong estrogenic activities, resulting in decreased fertility, increased fetal resorption, and changes in the weight of endocrine glands and serum hormone levels [10]. These compounds have a high relative binding affinity for estrogen receptors (ERs) and exhibit high transactivation activity [11], acting through ERs to activate the transcription of estrogen-responsive genes both in vivo [12,13] and in vitro [14]. ZEN also increased the expression of GRP78 and CCAAT/enhancer binding protein homologous protein (CHOP)—two ER stress-related marker genes [15]. ZEN could induce AIF- and ROS-mediated cell death through p53-and MAPK-dependent signaling pathways [16]. Moreover, its main metabolites, α-ZOL and β-ZOL, could induce loss of mitochondrial membrane potential (MMP), mitochondrial changes in Bcl-2 and Bax proteins, and cytoplasmic release of cytochrome c and apoptosis-inducing factor [17].

Furthermore, concern is increasing about exposure to mycotoxin mixtures because of their natural co-occurrence [18]. Unfortunately, the toxicity of mycotoxins in combination cannot always be predicted based upon their individual toxicities [19]. Previous study showed the simultaneous presence of mycotoxins in food commodities and diet may be more toxic than the presence of one mycotoxin alone [20]. The prima facie conclusion that multiple exposure may lead to additive, synergistic or antagonist toxic effects is insufficient to establish either the nature of combined effects or the relative potencies of these toxins [21]. Therefore, more studies focused on changes to cellular biological systems (e.g., genomics, transcriptomics, proteomics, and metabolomics) caused by co-exposure to mycotoxins are indeed necessary.

Unlike genes and proteins, whose net function is subject to epigenetic regulation and/or post-translational modification, metabolites are the end products of cellular regulatory processes, with the strongest correlation to phenotype, and whose levels can be regarded as the ultimate response of biological systems to genetic and/or environmental changes [22,23]. Metabolomics represents the global assessment of metabolites in a biological sample and reports the closest information to the phenotype of the biological system under study [24]. A metabolome is defined as the full complement of small-molecule metabolites found in a specific cell, organ, or organism [25], and is an excellent indicator of phenotypic heterogeneity. In turn, heterogeneity has been recognized as a key factor in rare-cell survival when populations are subjected to major chemical or environmental challenges [26].

The present study is to establish a GC-MS-based, complete metabolome method (serum-metabolome and liver-metabolome) to evaluate biological systems changes, as induced by DON and ZEN in mice. Applying the results, the relationship between combined toxic effects, in terms of additive, antagonistic or synergistic toxicity, and the metabolic profiling is assessed.

## 2. Results and Discussion

### 2.1. The Analysis of Blood Biochemical Indexes

**Figure 1:**
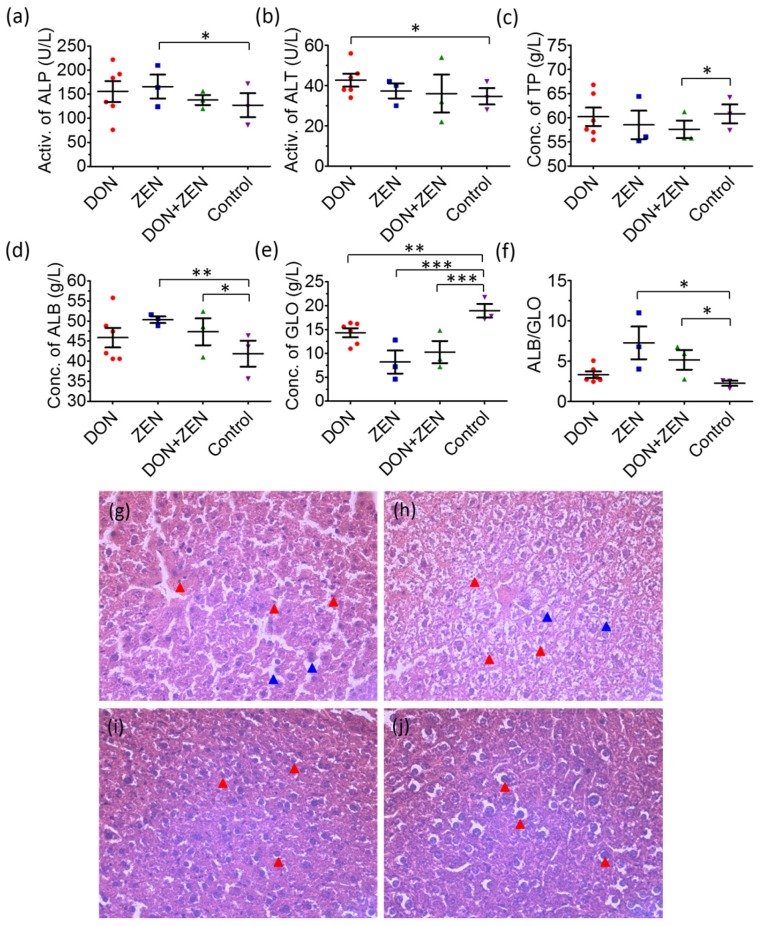
Scatter plots for serum levels of (**a**) Alkaline phosphatase (ALP); (**b**) alanine transaminase (ALT); (**c**) total protein (TP); (**d**) albumin (ALB); (**e**) globulin (GLO); and (**f**) ALB/GLO of mice treated with 2 mg/kg deoxynivalenol (DON), 20 mg/kg zearalenone (ZEN), and combined DON + ZEN with final concentration 2 mg/kg DON and 20 mg/kg ZEN, for 21 days. * for *p* value < 0.05; ** for *p* value < 0.01, and *** for *p* value < 0.001. Histopathological examination of control and mycotoxins-treated liver tissues by hematoxylin and eosin (HE) staining; (**g**) Liver of 2 mg/kg DON; and (**h**) liver of 20 mg/kg ZEN-treated mice for 21 days, showing epithelial cell (blue arrow) and inflammatory cell infiltration (red arrow); (**i**) Liver of combined DON + ZEN (final concentration 2 mg/kg DON and 20 mg/kg ZEN) treated mice for 21 days, showing slight inflammatory cell infiltration; (**j**) Control mice with normal livers. Magnification times: 200×.

Blood alkaline phosphatase (ALP) and alanine transaminase (ALT) levels are the most frequently reliable biomarkers of liver injury. Individual DON and ZEN exposure caused a significant increase in enzyme activities of serum ALP and ALT, which were decreased to normal levels in the combined DON + ZEN group, in Figure 1. These two enzymes are normally localized in the liver cytoplasm and are released into circulation after liver damage. Therefore, the increased levels of serum ALP and ALT in the individual DON and ZEN group might predict the hepatic injuries [27]. The decrease of ALP and ALT in the combined DON + ZEN group suggested the recovery of livers. The responses of total protein (TP) to mycotoxins exposure, however, were slightly different: the combined DON + ZEN seems to strengthen the toxicity of individual DON and ZEN. The albumin (ALB) and globulin (GLO) levels showed a slightly decreasing toxicity trend comparing combined DON + ZEN with individual DON and ZEN. However, the rate of ALB/GLO, for diagnosing kidney disease or liver disease, became moderated in the combined DON + ZEN group.

Liver tissue was hematoxylin and eosin (HE) stained for histopathological examination [28]. As shown in Figure 1, in livers from the individual DON- and ZEN-treated mice for 21 days, obvious cell necrosis was detected in midzonal and pericentral regions of the liver lobule accompanied by inflammatory infiltration of portal canal, and local cells exhibited nuclear fragmentation. The livers of combined DON + ZEN dosed mice presented invisible cell necrosis, which suggested that liver tissues had relatively weaker damage. Although, the liver tissue coefficient (Figure S1) and body weight monitor results (Figure S2) revealed no significant differences, expect for the slight body weight decrease in DON group.

According to results from the blood biochemical indexes and histopathological examination, the combined DON and ZEN may lead to an “antagonistic effect” in the liver toxicity related to metabolic changes in mice.

### 2.2. Multivariate Statistical Analysis

**Figure 2:**
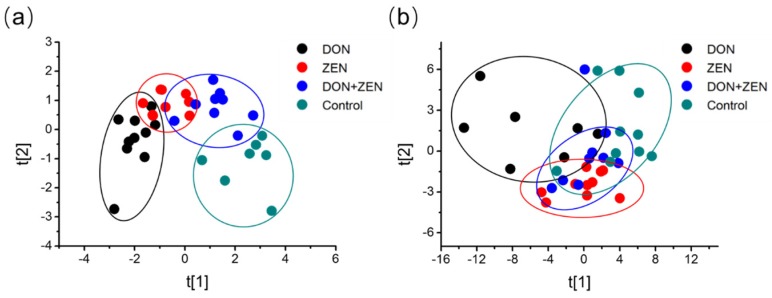
Non-supervised Principal Component Analysis (PCA). Five-week-old mice were treated with 2 mg/kg DON, 20 mg/kg ZEN, or combined DON and ZEN, with final concentration 2 mg/kg DON, 20 mg/kg ZEN, for 21 days. PCA representation of major sources of metabolites variability through a non-targeted analysis by GC-MS to monitor metabolic changes during the (**a**) serum (*R^2^X* = 0.37, *Q^2^* = 0.23) and (**b**) liver invasion (*R^2^X* = 0.38, *Q^2^* = 0.20). Data points represent four technical replicates from two independent experiments (biological replicates; *n* = 7–10) injected randomly into the GC-MS. The signals corresponding to different treatments were compared after treatment of log transformation and Pareto scaling.

Distinct spectral phenotypes were readily observed in the metabolites of the serum samples and liver tissue samples collected from control and mycotoxins-treated mice. Principle component analysis (PCA) was performed and the score plots of the control and treatment groups are shown in Figure 2, and the samples in the score plots of serum and liver tissue metabolomes were within the 95% Hotelling T2 ellipse. The PCA score plots for mycotoxins-treated groups are represented as a cloud of points in a multidimensional space with an arix for each of the components, which was an unsupervised classification for more realistically displaying the matrix of serum samples and liver tissue samples. The metabolic profiles in the four groups of serum samples, in Figure 2a, showed that the difference between the four groups were obvious after 21 days of mycotoxins treatment. Further, the distinction, made by the Simca-P software, implied that these three dosed groups, DON group, ZEN group, and DON + ZEN group, might have different damage severity on the mice’s biosystem, at least on the metabolic profiling in serum. Although, the “Mahalanobis distance” was not calculated accurately here, the DON-treated group and ZEN-treated group revealed more significant distinction as compared with the control group and the combined “DON + ZEN” group.

However, the metabolic profiles after 21 days of mycotoxins treatment did not show special obvious separations in the liver tissue sample, as shown in Figure 2b. ZEN is an estrogen-like mycotoxin, so the target organ for the animal's reproductive organs [29], and DON does not have a fixed target organ. Moreover, liver plays a major role in metabolism with numerous functions, particularly for glycogenolysis and detoxification, and was a key tissue for mycotoxins metabolism. The three treatment groups revealed obvious differences compared to the control group, in the unsupervised PCA classification. The DON-treated group showed a slight distinction from the ZEN-treated group and the DON + ZEN group.

This phenomenon, to some extent, indicated the “antagonistic effect” of DON and ZEN in liver metabolism of the mice.

### 2.3. Metabolites Screening

**Figure 3:**
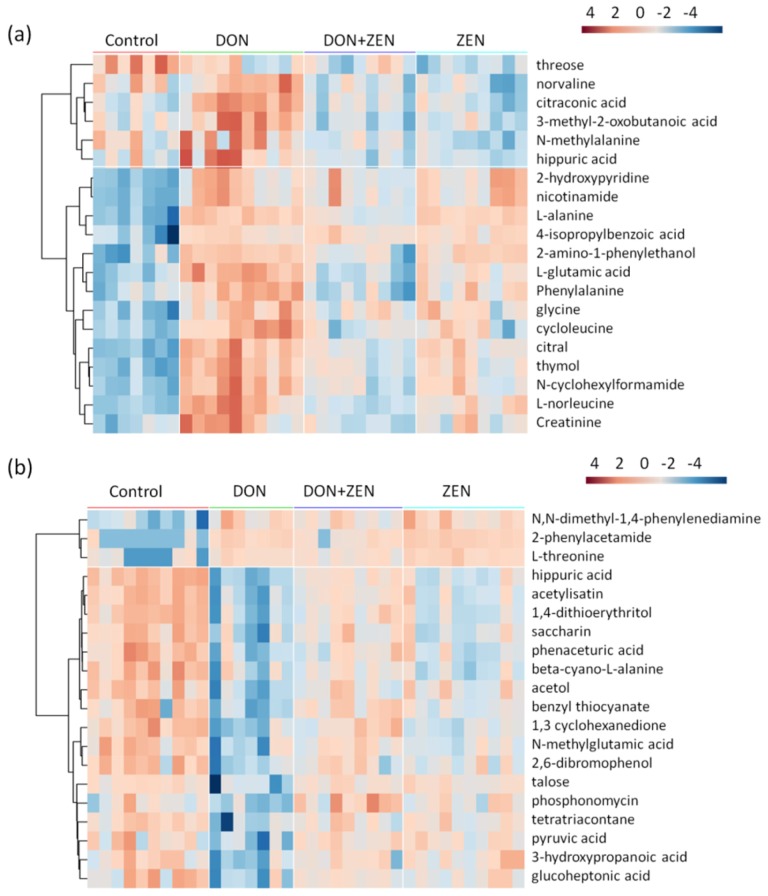
Heat maps, generated by hierarchical Pearson clustering, of the first 20 metabolites, significant from ANOVA, in the serum-metabolome (**a**) and liver-metabolome (**b**). The data set was screened using *t*-test *p* value < 0.05, variable importance for the projection (VIP) value > 1, similarity value > 700. Five-week-old mice were treated with 2 mg/kg DON, 20 mg/kg ZEN, or combined DON and ZEN, with final concentration 2 mg/kg DON, 20 mg/kg ZEN, for 21 days. Data points represent four technical replicates from two independent experiments (biological replicates; *n* = 7–10) injected randomly into the GC-MS. The signals corresponding to different treatments were compared after treatment of Log transformation and Pareto scaling, and the samples in the score plots of serum and liver tissue metabolomes were within the 95% Hotelling T2 ellipse.

Orthogonal partial least squares discriminant analysis (OPLS-DA) is an extension of PLS-DA, which is based on splitting the variations of variables into two parts: variation correlated to response and variation not correlated to response. Separating variation in this way aids interpretation of the model and identification of important variables. OPLS-DA, a supervised analysis technique, was employed to divide the different groups of serum samples and liver tissue samples. The metabolites selected for heatmap were based on the principles of the variable importance for the projection (VIP) value larger than 1 and the *t*-test at the 95% level. To better display the metabolites’ concentration discrepancy in the four groups of serum samples and liver tissue samples, only the first 20 metabolites were chosen for hierarchical Pearson clustering, as shown in Figure 3. The first 20 metabolites in serum samples were divided into 6 decreasing trend and 14 increasing trend, in Figure 3a; in liver tissue group, 3 up-regulated metabolites and 17 down-regulated metabolites, in Figure 3b. However, it could be obviously noticed that the color distribution in control group are similar with that in DON + ZEN group, in both serum group and liver tissue group, which indicated that the combined DON and ZEN might palliate the toxicity, compared with individual DON and individual ZEN. These data again support the hypothesis that the combined DON and ZEN can lead to an “antagonistic effect” in the metabolism of mice. To determine whether it is a “synergistic effect” or an “antagonistic effect” in the “biological metabolism” of mice, the metabolic pathway study was implemented.

### 2.4. Metabolic Pathway Analysis

**Figure 4:**
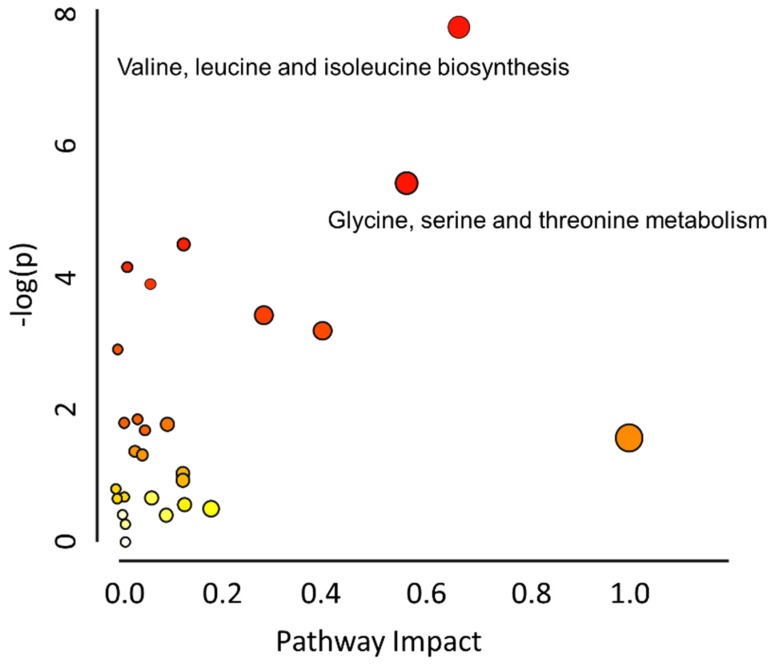
The pathway analysis of the identified metabolites affected in the three treatment groups. Based on the selected metabolites, the global metabolic disorders of the most relevant pathways induced by adenine were revealed using the MetaboAnalyst 3.0. Here, the x-axis represents the pathway impact and the y-axis represents the pathway enrichment. Larger sizes and darker colors represent higher pathway enrichment and higher pathway impact values.

**Figure 5:**
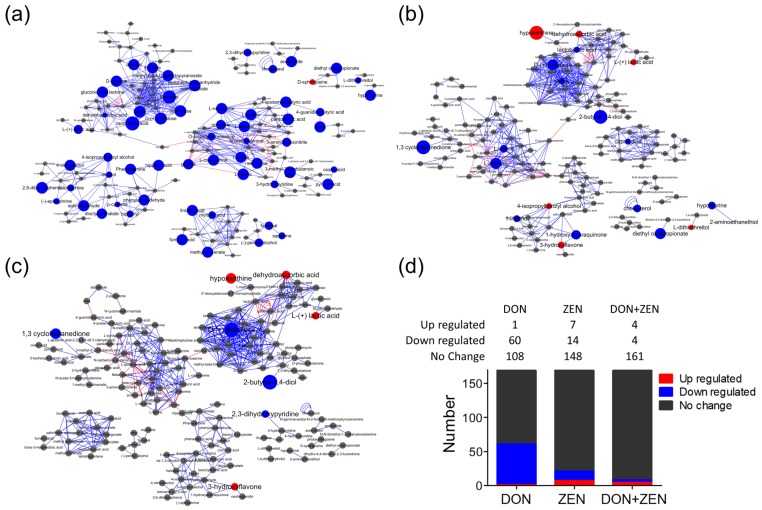
The pathway mapping of metabolites detected in liver of mice treated with (**a**) 2 mg/kg DON; (**b**) 20 mg/kg ZEN; and (**c**) DON + ZEN with final concentration of 2 mg/kg DON and 20 mg/kg ZEN, for 21 days. The metabolic pathways were generated through MetaMapp, and drew by CytoScape. The red balls represent increasing metabolites compared with control group, blue balls represent decreasing, and grey balls represent the metabolites with insignificant change, based on the fold change direction calculated by MetaMapp. The red line for the relationship between the two metabolites, which had been included in KEGG, and the blue line for the relationship between of two metabolites, which were not included in KEGG, but the connected metabolites had a similar functional group or similar chemical structure, judged by PubChem. The diameter of the ball was related to the fold change value and *t*-test p value calculated by MetaBox. (**d**) The change trend statistics analysis of the metabolites in the three treatment groups, DON/control group, ZEN/control group, and “DON + ZEN”/control group.

The pathway mapping was based on the KEGG web calculated by MetaboAnalyst, and only the recorded pathways were taken in to calculation, as shown in Figure 4. Valine, leucine and isoleucine biosynthesis, and glycine, serine and threonine metabolism were regarded as the first two affected pathways. The *O*-glycosyl compounds, like lactulose, d-trehalose talose, sophorose, etc., which had significant fold change and *t*-test result, could not be mapped in here. However, MetaMapp could calculate the relationship between the metabolites based on their chemical structure and functional groups classified by the PubChem [30]. Figure 5 showed metabolic pathway analysis of the three treatment groups, DON/control group, ZEN/control group, and “DON + ZEN”/control group, which were generated through MetaMapp, and drawn by CytoScape. In this research, with brief statistical analysis, the toxic effect on metabolic pathway become clear, among the total 169 identified metabolites, the number of changed metabolites is mostly found in DON treatment group (1 up-regulated and 60 down-regulated), and rarely happened in combined DON + ZEN group (4 up-regulated and 4 down-regulated), as shown in Figure 5d, which indicated that the combined DON and ZEN decreased the individual toxicity of DON or ZEN, revealing an “antagonistic effect” in liver tissue metabolism.

**Figure 6:**
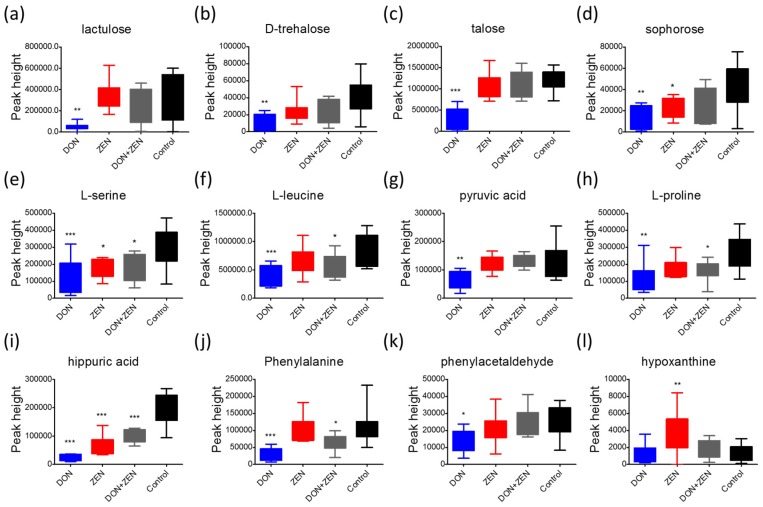
The peak height comparison of representative metabolites in four treatment groups, DON group (blue), ZEN group (red), DON + ZEN group (grey) and control group (black), whose VIP value > 1, *p* < 0.05, and similarity value > 700 were mapped with Fiehn library identified by MS DIAL software. The Dunett’s multiple comparison test was implemented for statistical analysis of the significance between the treatment groups (DON group, ZEN group, and DON + ZEN group) and the control group. * for *p* < 0.05, ** for *p* < 0.01%, and *** for *p* value < 0.001. (**a**) lactulose, (**b**) D-trehalose, (**c**) talose, (**d**) sophorose, (**e**) L-serine, (**f**) L-leucine, (**g**) pyruvic acid, (**h**) L-proline, (**i**) hippuric acid, (**j**) phenylalanine, (**k**) phenylacetaldehyde, (**l**) hypoxanthine.

As previously reported, DON affects the metabolic pathways of oxidative metabolism [31], glycolysis [32], and glutaminolysis [33], which preferentially fuel the cell fate decisions and effector functions of cells [34]. Interestingly, glycolysis or gluconeogenesis, starch and sucrose metabolism, and galactose metabolism were the significantly influenced glycometabolism of liver tissue induced in DON group, Figure 5a, indirectly involving lactulose, d-trehalose talose, sophorose, etc., belonging to the class of organic compounds known as *O*-glycosyl compounds, in Figure 6. These are glycosides in which a sugar group is bonded through one carbon to another group via an *O*-glycosidic bond. Additionally, lactulose is a synthetic disaccharide used in the treatment of constipation and hepatic encephalopathy. A proteomic analysis of the immunomodulatory effects, a down-regulation of transketolase (TK) and triosepho-sphate isomerase (TIM) induced by DON, illustrated that DON can produce the impairment of glycolysis (and gluconeogenesis) and, subsequently, lead to reduced energy generation [35]. Only d-trehalose behaved abnormally in the ZEN treatment group in Figure 5b or the combined DON + ZEN treatment group in Figure 5c, in glucose metabolism, which also indicated that the DON and ZEN had an “antagonistic effect” on glucose metabolism of liver tissue.

Amino acids play important roles in many metabolic pathways as basic substrates and as regulators [36]. A report [37] of gene pathway analysis indicated that DON down-regulated genes involved in glycolysis/gluconeogenesis, glutathione, glycosphingolipid biosynthesis, glycosaminoglycan biosynthesis, and the metabolism of many important chemicals such as galactose, starch, sucrose, fructose, mannose, pyruvate fatty acid, valine, leucine and isoleucine. These findings suggested that DON can strongly regulate the networks of genes that are predicted to change the balance of cell homeostasis. In our study, the metabolites that demonstrated statistically significant fold changes in DON treatment group included l-serine, l-leucine, pyruvic acid, l-proline, etc., as shown in Figure 6, which mainly involved the metabolism of glycine, serine and threonine, and biosynthesis of valine, leucine and isoleucine. However, only l-alanine and l-valine behaved abnormally in the liver metabolism of mice found in ZEN treatment group, in Figure 5b, and the such phenomenon seems less serious in the combined DON + ZEN treatment group as shown in Figure 5c. Some the precursor metabolites of amino acid, glycine, tyrosine, revealed inconsistent with the combined effect, e.g., hippuric acid and phenylalanine in Figure 6. In addition, the purine derivative, hypoxanthine, which increased in ZEN group, behaved well in individual DON and combined DON + ZEN group. All the metabolites with significant fold change and acceptable *t*-test results were considered for pathways analysis, which were shown in Table S1.

It was not expected that the metabolites in serum had an absolute positive correlation with those in liver tissue, because blood is an integrative biofluid that incorporates the functions and phenotypes of many different parts of the body in a single sample, a “metabolic footprint” of tissue metabolism. However, this complexity can dilute small metabolic changes from a specific part of the body, and in these cases, tissues may be appropriate for knowledge discovery. Hence, blood provides an appropriate overview of many areas of metabolism in the body. The identified metabolites were processed for the pathway mapping, as shown in Figure S3, although no metabolic pathway theoretically exited in blood. With mapping with KEGG, and chemical structure analysis with PubChem by MetaMapp, the integrated metabolic pathway revealed that the number of up-regulated or down-regulated metabolites decreased significantly in the combined DON + ZEN treatment group, comparing with individual DON or ZEN treatment group, which further verified the mentioned hypothesis that the combined DON and ZEN treatment has an “antagonistic effect” on the mouse metabolism.

## 3. Conclusions

It could be concluded from our results that the combined DON and ZEN treatment for 21 days revealed an obvious “antagonistic effect” in the metabolism of mice, as shown by their distinguished metabolic profiling from individual DON and ZEN treatment groups. The liver tissue histopathological examination and serum biochemical indexes detection found that the combined DON + ZEN treatment had slightly weaker toxicity than individual DON and ZEN treatment groups. The integrated metabolic pathways suggested that the combined DON + ZEN treatment weakens the toxic damage on the liver biological metabolism of mice, valine, leucine and isoleucine biosynthesis, and glycine, serine and threonine metabolism and *O*-glycosyl compounds-related glucose metabolism.

## 4. Materials and Methods

### 4.1. Chemicals and Reagents

Deoxynivalenol (DON), zearalenone (ZEN), methoxyamine hydrochloride and *N*-Methyl-*N*-(trimethylsilyl)trifluoroacetamide (MSTFA) were purchased from Sigma-Aldrich Co. LLC., Shanghai, China. The phosphate buffer solution (PBS, 0.1 M K_2_HPO_4_/NaH_2_PO_4_, pH 7.29) used in this study was obtained from Beyotime biotechnology, China. All other chemicals used were of HPLC grade. Deionized water used for all experiments was purified with a Milli-Q system (Millipore, Billerica, MA, USA).

### 4.2. Animal Handling and Treatment

All animals received humane care, and study protocols complied with the ethical guidelines of the European Community guidelines (Directive 2010/63/EU). The Jiangsu Science and Technology Department (SYXK [Su]2012-0002) approved all experimental procedures on 7 April 2016, project ID: JN.No20160322-20160701 [25]. A total of 40 Kunming (KM) mice (half male and half female), 5 weeks of age, were obtained from the animal facility of the institute and were acclimatized for 7 days in polypropylene cages at room temperature, 22 ± 2 °C, and relative humidity of 50% ± 10%. The light cycle was maintained at 12 h of light and 12 h of darkness. Food and water were provided ad libitum. Mice were randomly divided into four groups with an equal number of animals (*n* = 10 in each group, 5 male mice and 5 female mice, feeding separately) treated with 2 mg/kg DON, 20 mg/kg ZEN and the mixture of DON and ZEN (2 mg/kg DON, 20 mg/kg ZEN), through intragastric administration for three weeks; the solvent used was 90% saline and 10% ethanol. The mice of control group were treated with 90% saline and 10% ethanol. All animal handling and experimental protocols were performed in strict accordance with the guidelines of the Institutional Animal Ethics Committee. Blood was collected from eye, then mice were sacrificed. Finally, livers were dissected, quickly freeze clamped in liquid nitrogen and stored at −80 °C until further processing.

### 4.3. Metabolite Extraction

Freeze-dried liver powder (20 mg) was extracted with 1 mL of 50% cold acetonitrile (ACN) by a homogenizer, five times operated at 5500 rpm for 20 s with 30 s intervals [38]. The homogenized tissue samples were implemented with ultrasonic treatment for 5 min, then centrifuged in a MicRocl 21R Multi speed Centrifuge (Thermo, Conroe, Texas, USA) at 13,000× *g* at 4 °C for 15 min. 100 μL serum was extracted with 1 mL extraction solution (acetonitrile, isopropanol and water in proportions 3:3:2) [39]. The samples were then vortexed for 10 s, centrifuge samples for 13,000× *g* at 4 °C for 15 min. Finally, 300 μL metabolites solution supernatant (liver or serum) was transferred to a new 2 mL GC/MS glass vial. Quality control: An equal volume of 10 μL (based on the number of samples) was taken from each sample into the 2 mL GC-MS glass vial as a mixed sample [40]. Following centrifugation, the flow-through was saved and then dried for several hours in a Savant High Capacity Speedvac Plus Concentrator (Thermo Fisher, San Jose, CA, USA). Eighty microliters of methoxylamine hydrochloride (dissolved in pyridine, final concentration of 20 mg/mL) was added into dried metabolites, incubation at 80 °C for 20 min in an oven after mixing and sealing. The lid was opened and 100 μL MSTFA (containing 1% TCMS, *v*/*v*) added into each sample before being sealed again and incubated at 70 °C for an hour. Finally, the mix wells were waiting for GC-MS analysis [41]. Blood biochemical indexes detection was implemented in the Fourth People’s Hospital of Wuxi City, China, on the Hitachi 7020 blood biochemical analyzer.

### 4.4. GC/MS Analysis

GC-MS analysis was performed using a Shimadzu QP2010 Ultra gas chromatograph system coupled with mass spectrometer. The system utilized an Rxi-5Sil MS column (30 m × 250 μm inner diameter, 0.25 μm film thickness; Restek, Bellefonte, PA, USA). A 1 μL aliquot of the sample solution was injected into a splitless injector. Helium was used as the carrier gas, the front inlet purge flow was 3 mL·min^−1^ and the gas flow rate through the column was 20 mL·min^−1^. The initial temperature was kept at 70 °C for 1 min, then raised to 280 °C at a rate of 6 °C·min^−1^, and maintained for 5 min at 280 °C. The injection, transfer line and ion source temperatures were 280 °C, 280 °C, and 250 °C, respectively. The energy was −70 eV in electron impact mode. The mass spectrometry data were acquired in full-scan mode with the *m*/*z* range of 50–600 at a rate of 20 spectra per second after a solvent delay of 366 s.

### 4.5. Metabolite Profiling Analysis

Raw data was converted to “mzXML” format with the GCMS PostRun from Shimadzu company, and then converted to “abf” format with the ABF converter. The MS DIAL with Fiehn library were used for raw peaks exaction (files of “abf” format), the data baseline filtering and calibration of the baseline, peak alignment, deconvolution analysis, peak identification and integration of the peak height [42]. The example can be found in Figures S4 and S5, using metabolites lactulose. Average peak width of 20 scan and minimum peak height of 10,000 amplitudes was applied for peak detection, and sigma window value of 0.5, EI spectra cut-off of 5000 amplitudes was implemented for deconvolution. For identification setting, the retention time tolerance was 0.5 min, the *m*/*z* tolerance was 0.5 Da, the EI similarity cut-off was 70%, and the identification score cut-off was 70%. In the alignment parameters setting process, the retention time tolerance was 0.075 min, and retention time factor was 0.5.

### 4.6. Multivariate Analysis

Statistical data analysis was performed using Simca-P 14+, which was used for the principal component analysis (PCA) and orthogonal projection to latent structures-discriminant analysis (OPLS-DA). Heatmap analysis was performed with Metaboanalyst 3.0 (Montréal, QC, Canada), a web-based tool for pathway analysis and visualization metabolomics. The pathway mapping was analyzed with MetaMapp, and drawn by CytoScape 3.4.0. (Boston, MA, USA) GraphPad Prism (La Jolla, CA, USA), R i386 3.3.1, and Origin were used for the statistical analysis, including one-way ANOVA test (Dunnett’s multiple comparison test) and *t*-test.

## Supplementary Materials

The following are available online at www.mdpi.com/2072-6651/9/1/28/s1, Figure S1: The organ coefficient of liver in the four groups, DON (2 mg/kg), ZEN (20 mg/kg), DON + ZEN (final concentration DON 2 mg/kg, ZEN 20 mg/kg) and control groups, recorded at the 21st day, when mice were sacrificed, Figure S2: The time series of body weight of mice in the four groups, DON (2 mg/kg), ZEN (20 mg/kg), DON + ZEN (final concentration DON 2 mg/kg, ZEN 20 mg/kg) and control groups, recording from the 1st day to 17th day; the final 4 days was not monitored. * for *p* value < 0.5%, Figure S3: The pathway mapping of metabolites detected in urine of mice treated with (**a**) 2 mg/kg DON; (**b**) 20 mg/kg ZEN; and (**c**) combined DON + ZEN with final concentration 2 mg/kg DON and 20 mg/kg ZEN for 21 days. The metabolic pathway was generated through MetaMapp, and drew by CytoScape. The red balls represent increasing metabolites comparing with control group, blue represent decreasing, and green represent the metabolites with insignificant change, based on the fold change direction calculated by MetaMapp. The red line for the relationship between the two metabolites, which had been included in KEGG, and the blue line for the relationship between of two metabolites, which were not included in KEGG, but the connected metabolites had a similar functional group or similar chemical structure, judged by PubChem. The diameter of ball was related with the fold change value and *t*-test *p* value calculated by MetaBox, Figure S4: (**a**) The raw EI spectrum of lactulose extracted from GC-MS; (**b**) the deconvoluted EI spectrum of lactulose processed by MS DIAL software, with average peak weight 20 scan (5 Hz (scan rate) × 4 s (average a peak time)). (**c**) The raw EI chromatograms of lactulose extracted from GC-MS; (**d**) the deconvoluted EI chromatograms of lactulose, Figure S5: (**a**) the peaks alignment of lactulose in the 40 liver samples, with time error 0.1 min. (**b**) The library mapping of the experimental deconvoluted EI spectrum of lactulose with the standard spectrum of lactulose. (**c**) The chemical structure of lactulose identified through Fiehn library. (**d**) The total ion chromatograms of one liver sample; the red marked peaks, in the zoom-in chromatograms, is lactulose.
