# Supplementary material for: The Antagonistic Effect of Mycotoxins Deoxynivalenol and Zearalenone on Metabolic Profiling in Serum and Liver of Mice
Source: Toxins (Basel). 2017 Jan 10;9(1):28. doi: 10.3390/toxins9010028 (PMC5308260; doi:10.3390/toxins9010028)
Supplement: Supplementary file 1 [file toxins-09-00028-s001.pdf]

Supplementary Materials: The Antagonistic Effect of Mycotoxins Deoxynivalenol and Zearalenone on Metabolic Profiling in Serum and Liver of Mice

Jian Ji, Pei Zhu, Fangchao Cui, Fuwei Pi, Yinzhi Zhang, Yun Li, Jiasheng Wang and Xiulan Sun

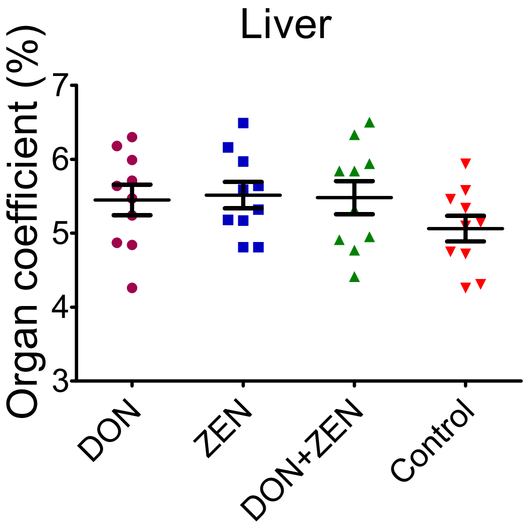

Figure S1. The organ coefficient of liver in the four groups, DON (2 mg/kg), ZEN (20 mg/kg), DON + ZEN (final concentration DON 2 mg/kg, ZEN 20 mg/kg) and control groups, recorded at the 21st day, when mice were sacrificed.

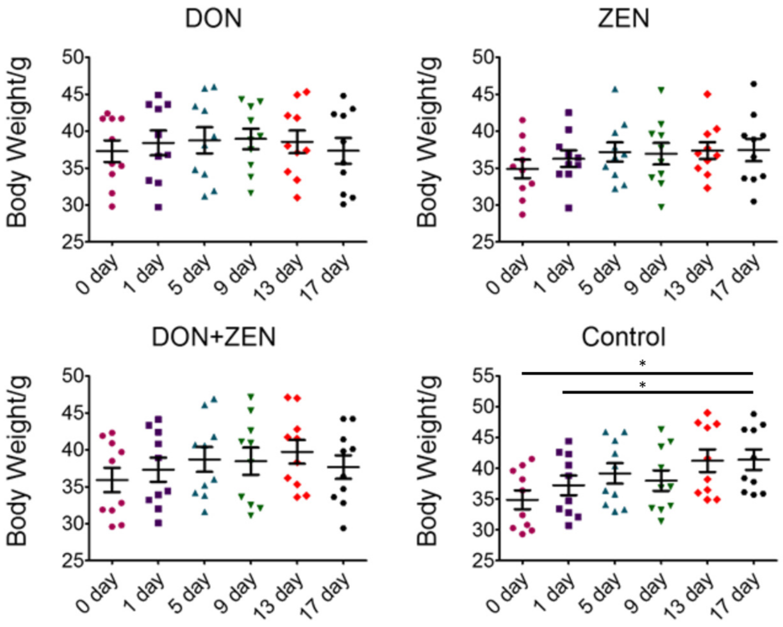

Figure S2. The time series of body weight of mice in the four groups, DON (2 mg/kg), ZEN (20 mg/kg), DON + ZEN (final concentration DON 2 mg/kg, ZEN 20 mg/kg) and control groups, recording from the 1st day to 17th day; the final 4 days was not monitored. * for p value < 0.5%.

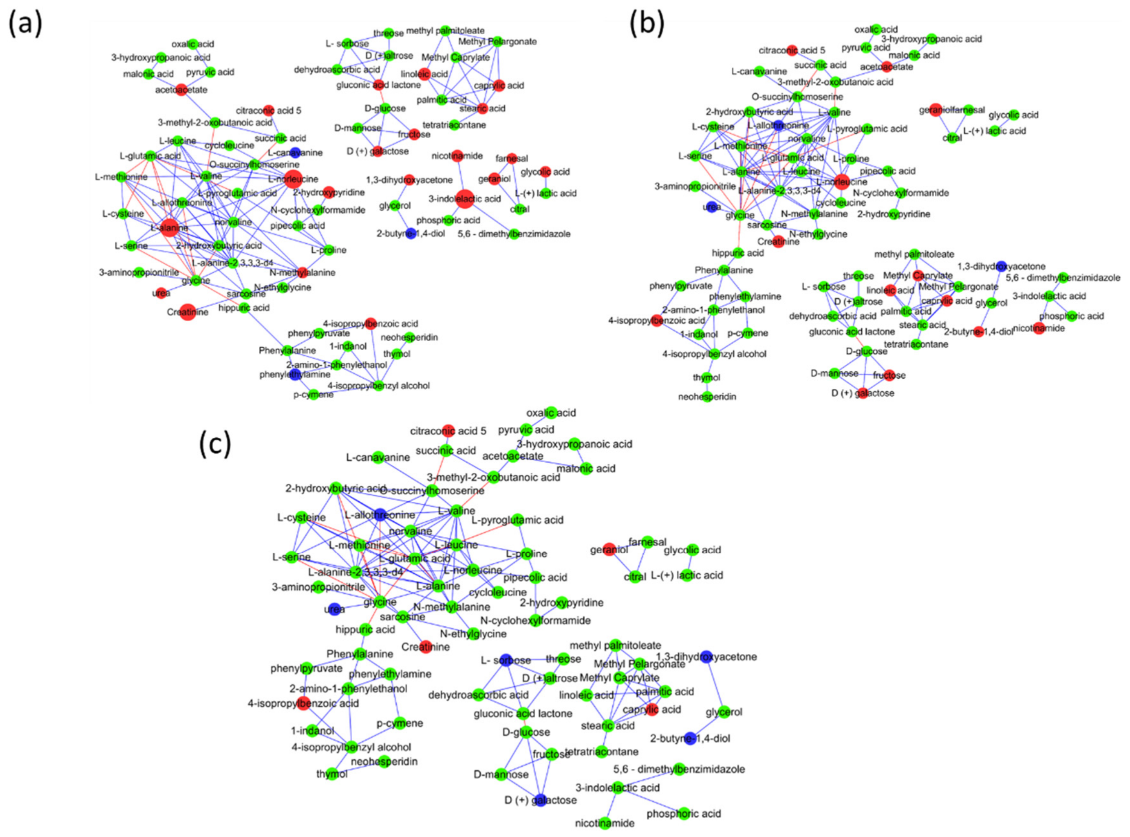

Figure S3. The pathway mapping of metabolites detected in urine of mice treated with (**a**) 2 mg/kg DON; (**b**) 20 mg/kg ZEN; and (**c**) combined DON + ZEN with final concentration 2 mg/kg DON and 20 mg/kg ZEN for 21 days. The metabolic pathway was generated through MetaMapp, and drew by CytoScape. The red balls represent increasing metabolites comparing with control group, blue represent decreasing, and green represent the metabolites with insignificant change, based on the fold change direction calculated by MetaMapp. The red line for the relationship between the two metabolites, which had been included in KEGG, and the blue line for the relationship between of two metabolites, which were not included in KEGG, but the connected metabolites had a similar functional group or similar chemical structure, judged by PubChem. The diameter of ball was related with the fold change value and *t*-test *p* value calculated by MetaBox.

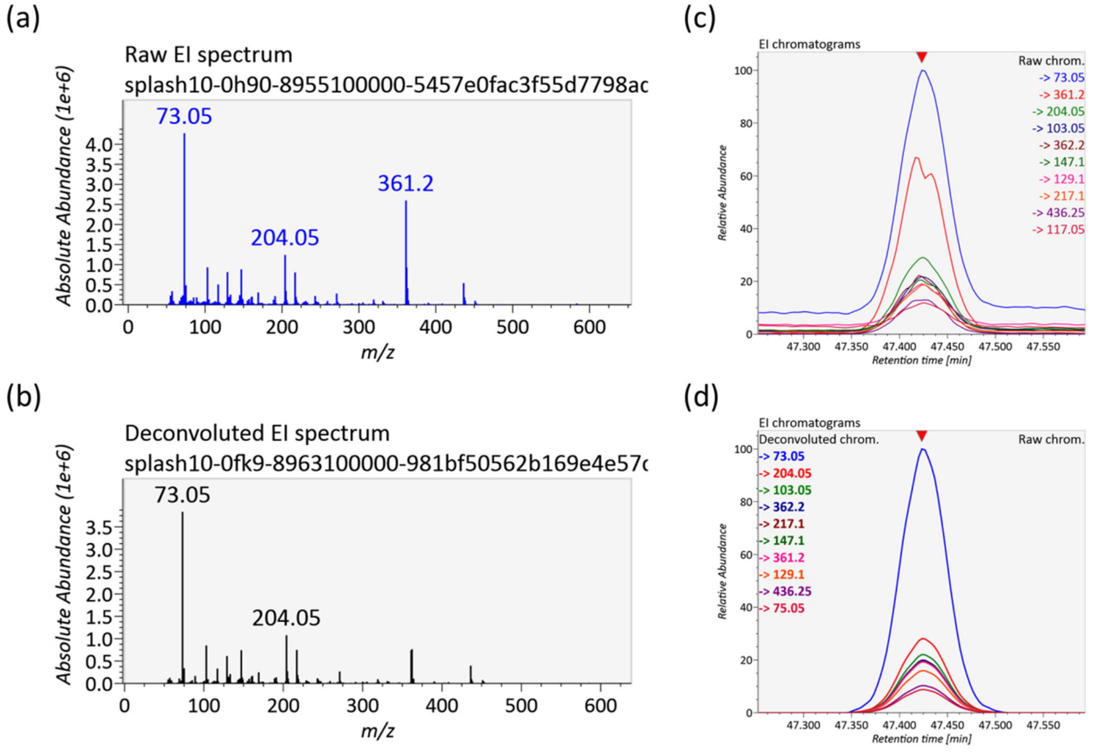

Figure S4. (**a**) The raw EI spectrum of lactulose extracted from GC-MS; (**b**) the deconvoluted EI spectrum of lactulose processed by MS DIAL software, with average peak weight 20 scan (5 Hz (scan rate) × 4 s (average a peak time)). (**c**) The raw EI chromatograms of lactulose extracted from GC-MS; (**d**) the deconvoluted EI chromatograms of lactulose.

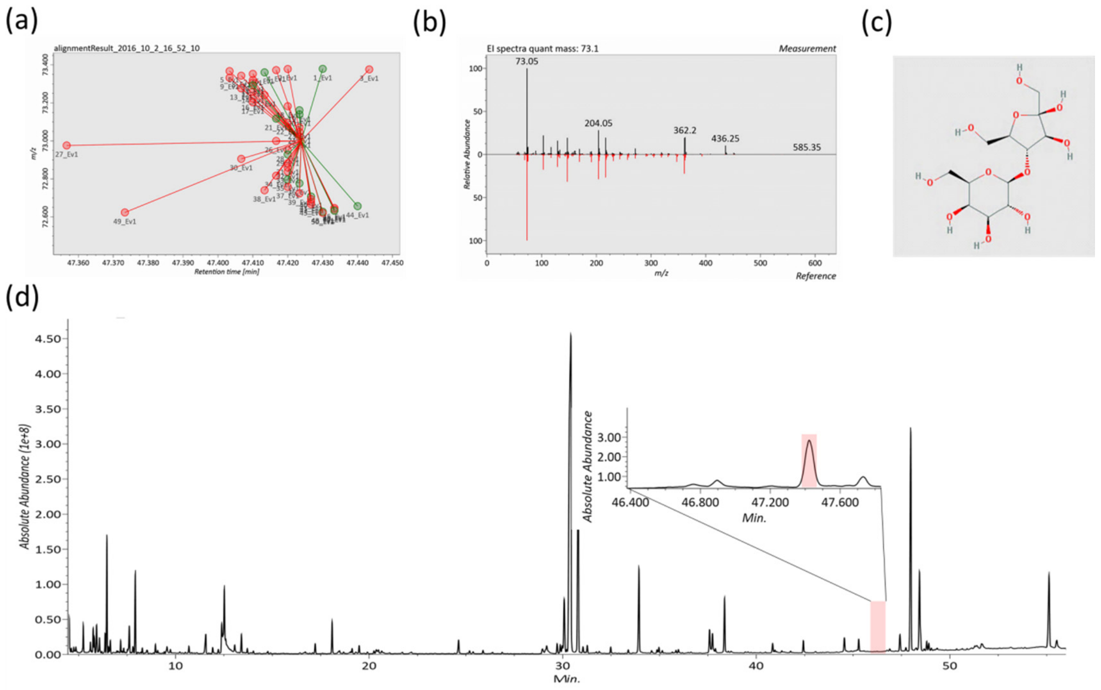

Figure S5. (**a**) the peaks alignment of lactulose in the 40 liver samples, with time error 0.1 min. (**b**) The library mapping of the experimental deconvoluted EI spectrum of lactulose with the standard spectrum of lactulose. (**c**) The chemical structure of lactulose identified through Fiehn library. (**d**) The total ion chromatograms of one liver sample; the red marked peaks, in the zoom-in chromatograms, is lactulose.
